# The Tumor Necrosis Factor Superfamily of Cytokines in the Inflammatory Myopathies: Potential Targets for Therapy

**DOI:** 10.1155/2012/369432

**Published:** 2011-10-23

**Authors:** Boel De Paepe, Kim K. Creus, Jan L. De Bleecker

**Affiliations:** Laboratory for Myopathology, Department of Neurology and Neuromuscular Reference Center, Ghent University Hospital, 9000 Ghent, Belgium

## Abstract

The idiopathic inflammatory myopathies (IM) represent a heterogeneous group of autoimmune diseases, of which dermatomyositis (DM), polymyositis (PM), and sporadic inclusion body myositis (IBM) are the most common. The crucial role played by tumor necrosis factor alpha (TNF**α**) in the IM has long been recognized. However, so far, 18 other members of the TNF superfamily have been characterized, and many of these have not yet received the attention they deserve. In this paper, we summarize current findings for all TNF cytokines in IM, pinpointing what we know already and where current knowledge fails. For each TNF family member, possibilities for treating inflammatory diseases in general and the IM in particular are explored.

## 1. Introduction

The idiopathic inflammatory myopathies (IM) are characterized by distinct immune effector mechanisms. Dermatomyositis (DM) is a complement-mediated endotheliopathy associated with perimysial inflammation and perifascicular muscle fiber atrophy. In polymyositis (PM) and sporadic inclusion body myositis (IBM), muscle fibers are injured by autoaggressive immune cells which predominantly infiltrate the endomysium [1]. Additional degenerative phenomena occur in IBM muscle, such as muscle fiber vacuolation and deposition of *β*-amyloid and other ectopically localized proteins [2]. Other less well-delineated IM include myositis-associated cancer or connective tissue diseases and immune-mediated necrotizing myopathy. Macrophages, dendritic cells (DCs), and T-cells are prominently present in muscle tissues of the different IM. In DM, large numbers of helper T-cells are found within the perimysial, often perivascular, inflammatory infiltrates. In PM and IBM, activated cytotoxic T-cells surround and invade nonnecrotic muscle fibers, while helper T-cells are found at more distant parts of the infiltrates. B-cell-mediated immunity is an important component of DM/PM pathogenesis, and autoantibodies can be detected in up to 70% of patients [3]. The most common is Jo-1, an antihistidyl-tRNA-synthetase, but more autoantibodies directed against aminoacyl-tRNA-synthetases or other muscle antigens are continuously being described. Autoantibody profiles are associated with clinical subsets of patients [4]. In IBM on the other hand, humoral autoimmunity is a more controversial subject. However, it has been established that IBM muscle contains large numbers of plasma cells and an environment permissive of ectopic lymphoneogenesis, which suggests the possibility of local maturation of B-cells and autoantibody production. Indeed, a recent report describes IBM-specific autoantibodies directed against a yet unidentified muscle antigen [5].

The important role played by cytokines in the IM has long been recognized [6]. In this respect, a key role for type I interferon (IFN)-mediated innate immunity has been shown in DM and PM [7, 8]. In this paper, the tumor necrosis factor (TNF) family will be systematically overviewed, discussing current knowledge on their involvement in the IM and exploring whether they could represent appropriate targets for future therapeutic intervention. TNF cytokines affect immune cell proliferation, differentiation, and survival. Up till now, 19 members have been identified in man, and they have been assigned systematic names starting from TNFSF1 to TNFSF18 based on the encoding genes. Ectodysplasin A (EDA) 1 and 2 have not been assigned systematic names and will not be discussed in this paper. Most TNF members are type II transmembrane proteins whose extracellular C-terminal TNF homology domain can be cleaved off by specific metalloproteinases, generating soluble cytokines. The TNF-like receptors (TNFR) are type I transmembrane proteins of which cystein-rich domains are the hallmark structural motif [9].

## 2. TNFSF2—TNF*α*

TNF*α* or cachectin, the prototypic member of the TNF family, is produced mainly by activated macrophages and T-cells. TNF*α* activates T-cells, B-cells, and macrophages and induces the expression of other cytokines and cell adhesion molecules through interaction with its receptor TNF-R55 (TNFR1). The alternative receptor TNF-R75 (TNFR2) has been shown to chiefly function as a concentrator at cellular surfaces, transferring the cytokine to TNF-R55 [10]. TNF*α* augments the activity of nuclear factor-*κ*B (NF-*κ*B) signaling pathways [11].

In the IM, TNF*α* is by far the most studied cytokine of its family. The endomysial and perimysial inflammatory cells express varying levels, with macrophages being the primary source of the cytokine. TNF*α* is also prominently expressed in blood vessel endothelial cells of DM tissues [12–14]. The soluble forms of the receptors TNF-R55 and TNF-R75 are increased in DM/PM sera [15]. TNF-R75 expression is notably increased near inflammatory infiltrates in all IM and on the perimysial and perifascicular blood vessel endothelium in DM even remote from inflammation [12]. Polymorphisms in the gene encoding TNF*α* have been linked to either an increased risk of, or protection against, the development of juvenile DM [16, 17].

Neutralization of TNF*α* is efficacious for treating several autoimmune diseases. The important catabolic role of TNF*α* as a regulator of the chronic inflammation associated with the IM has made it a therapeutic target for these diseases as well. Fortunately, knocking out TNF*α* appears relatively safe and does not seem to hamper skeletal muscle regeneration [18]. Four agents, that generate excellent results in rheumatoid arthritis (RA) and Crohn's disease, can be considered for IM patients: (1) a mouse/human chimeric anti-TNF*α* monoclonal antibody termed infliximab (Remicade), (2) a TNF*α*-neutralizing receptor fusion protein termed etanercept (Enbrel), (3) a humanized anti-TNF*α* monoclonal antibody termed adalimumab (Humira), and (4) the humanized polyethyleneglycol conjugated Fab′ anti-TNF*α* fragment certolizumab pegol (Cimzia). For the first two compounds, reports so far have revealed variable outcomes in IM patients. Trial results are summarized in Table 1 [19–26]. Several phase II clinical trials have been started up, but, in general, studies suffer from low inclusion rate and notably high drop-out rates mostly due to disease deterioration and adverse events. However, it appears that anti-TNF*α* treatment could be of benefit to a subset of IM patients. The identification of responsive patients remains difficult, as no specific marker has been identified yet that may predict the therapeutic outcome. 

## 3. Other TNF Members Already Investigated to Some Extent in the IM

### 3.1. TNFSF1/3—LT*α*/*β*

Lymphotoxins (LTs) are versatile cytokines. They are crucial for robust immune responses and are key elements required for lymphoid organogenesis and organization. LT*α*, also termed TNF*β*, is secreted as the homotrimer LT*α*3, or complexed on the cell surface with LT*β*, predominantly as LT*α*1*β*2 heterotrimers. LT*α* can bind to the receptor LT*β*R as well as to the receptors TNFR1 and TFNR2. LT*β* signals through LT*β*R ligation only.

It appears that LTs are important factors orchestrating sustained inflammation in the IM. LT*α* has been implicated in the cytotoxic response of CD8^+^ T-cells towards nonnecrotic muscle fibers in PM [27]. LT*β* is increased in muscle tissues of DM patients, where it localizes to blood vessels and intramuscular follicle-like structures. The latter contain large numbers of T-cells, B-cells, and DCs organized in functional compartments [28]. Recent data also show that LT*β* may well be an early marker for muscle disease [29]. 

LTs have been pinpointed as important targets for suppressing inflammation in autoimmune diseases. Studies showed that depletory monoclonal anti-LT*α* and the receptor antagonist LT*β*R:Ig inhibit disease in murine collagen-induced arthritis [30, 31]. In addition, administering LT*β*R:Ig inhibited T-cell-driven intestinal inflammation in murine inflammatory bowel disease [32]. In human RA, synovial LT*α* and LT*β* expression is elevated [33], but targeting the expression by administering LT*β*R:Ig failed to meet clinical end points in a phase IIb clinical trial. As TNF*α* and LT*α* share the receptors TNFR1 and TNFR2, strategies targeting these receptors influence the activities of both cytokines. Therefore, the therapeutic effects of competitive antagonists of TNFR1 and TNFR2, namely, etanercept and lenercept, are presumed to result from combined inhibition of TNF*α* and LT*α*.

### 3.2. TNFSF4—OX40L

The primary source of the transmembrane glycoprotein OX40L, also termed CD252 or gp34, are antigen presenting cells, and expression is further induced when B-cells, DCs, T-cells, and macrophages become activated. The cytokine promotes clonal expansion of T-cells, leading to long-term T-cell survival and enhanced memory T-cell development. The receptor OX40, also termed CD134, is expressed on activated T-cells, B-cells, and vascular endothelial cells. Proinflammatory cytokines, including TNF*α*, can further augment the expression of the receptor. The OX40/OX40L interaction provides a costimulatory signal for T-cells and enhances ongoing immune responses driven by either helper T-cell type 1 (Th1), Th2, or Th17 cells [34].

OX40 is present on mononuclear cells in the endomysium and at perivascular sites in PM muscle. OX40 positive cells are mostly CD4^+^ cells, few are CD8^+^ cytotoxic T-cells. Autoaggressive immune cells invading nonnecrotic muscle fibers are invariably OX40 negative [35]. OX40L expression has not yet been described in the IM.

Blocking OX40L has produced strong therapeutic effects in multiple animal models of autoimmune and inflammatory disease, which include inflammatory bowel disease [36] and arthritis [37]. Neutralizing antibodies to OX40L are currently being tested in phase II clinical trials for treating asthma.

### 3.3. TNFSF5—CD40L

CD40L, also termed CD154 or gp39, is expressed by activated T-cells, mainly on the CD4^+^ subsets. Its receptor CD40 is present on antigen presenting cells and on endothelial cells. CD40L positive T-cells activate monocytes and upregulate adhesion molecules and monocyte chemoattractant protein 1 (CCL2) production by blood vessel endothelial cells [38]. 

The CD40/CD40L system seems to be involved in the immunopathogenesis of the IM. A subset of inflammatory cells in IM tissues express CD40L, of which the majority are CD4^+^ cells. Also, part of the muscle fibers in PM/DM tissues express CD40. In vitro IFN*γ*-stimulation of myoblasts induces CD40 expression, leading to increased levels of IL-6, IL-8, IL-15, and CCL2 [39]. The induction of proinflammatory factors through the CD40/CD40L system could contribute to T-cell recruitment and activation found within IM muscle tissues.

CD40L/CD40 interaction engages antigen presenting cells, provokes B-cell responses and enhances the production of proinflammatory cytokines, pinpointing the interaction as an important regulatory mechanism in inflammatory disease. In murine collagen-induced arthritis, for example, an agonistic anti-CD40 antibody exacerbates disease [40], while a blocking anti-CD40L antibody protects against disease [41]. Phase I and II trials in humans have already been initiated, testing the effects of a humanized anti-CD40L antibody in inflammatory bowel disease [42]. However, the development of IDEC-131, another humanized monoclonal anti-CD40L, is no longer pursued after a placebo-controlled trial demonstrated no clinical activity in systemic lupus erythematosus (SLE) [43].

### 3.4. TNFSF6—FASL

FasL is expressed on activated T-cells and NK-cells. The cytokine comes in a 40 kDa membrane-bound and a 29 kDa-soluble variant. Its receptor Fas, also termed CD95 or apoptosis 1 (Apo1), is constitutively expressed on many cell types. An alternative soluble receptor termed decoy receptor 3 (DCR3) has been described, possibly serving to counteract Fas function. Fas ligation leads to a conformational change, which causes binding of death domain-containing adaptor proteins, subsequently activating caspases and nucleases.

A role for the Fas/FasL system in muscle damage is suspected. Proinflammatory in vitro conditions have been shown to induce apoptosis in muscle cells, a process that can be partially inhibited by an anti-FasL antibody [44]. However, apoptosis is not a prominent feature of IM, and data concerning Fas/FasL expression appear somewhat confusing. FasL was found absent from IM muscle tissue [45] or expressed only by some T-cells [27, 46]. Fas expression has been reported with very different frequencies, but the sarcolemma of regenerating muscle fibers seems to represent the main site of immunoreactivity. Also, some nonnecrotic invaded muscle fibers in PM/IBM and some atrophic perifascicular muscle fibers of DM are Fas positive [45, 47, 48]. Serological studies report unchanged FasL levels, while Fas levels were significantly higher in PM/DM patients compared to normal controls [49]. The peripheral blood of DM patients contains significantly lower percentages of regulatory T-cells, but the fraction of Fas positive cells is similar, indicating no increased susceptibility of regulatory T-cells to FasL-mediated apoptosis [50]. 

The involvement of Fas/FasL interactions in human inflammatory disease is complex. It has been shown that Fas/FasL deficiencies are associated with the accumulation of lymphocytes and establishment of autoimmune disease. Indeed, a number of inflammatory diseases seem to be associated with decreased serum levels of soluble FasL. Administering DCs overexpressing FasL resulted in protection against murine collagen-induced arthritis [51] possibly through elimination of autoreactive T-cells. Nonetheless, in RA synovial fluid, increased levels of soluble FasL have been found [52]. More research is needed to unravel the precise involvement of the cytokine in human diseases.

### 3.5. TNFSF7—CD27L

CD27L, also termed CD70, is expressed on T-cells, B-cells, and NK-cells. It is a T-cell costimulatory molecule whose expression is upregulated upon activation. CD27L regulates the formation of effector and memory T-cells and induces their secretion of cytokines. In the B-cell compartment, CD27L promotes differentiation into plasma cells and subsequent antibody production, commitment to memory B-cell responses, and the formation of germinal centers. The receptor CD27 is constitutively expressed on resting T-cells and is upregulated upon T-cell activation. In B-cells, CD27 expression is induced by antigen-receptor activation. 

Very limited data is currently available on CD27L expression in the IM. We do know that unlike in the series of SLE patients, CD27L is not increased on peripheral CD4^+^ cells of a single DM patient, who was included as a disease control in the study [53]. 

Under physiological conditions, expression of CD27L is restricted and transient in nature. Thus, CD27L offers a potential mechanism to selectively target only the activated cells of the immune system, B-cells in particular, potentially avoiding generalized immunosuppression and overt toxicity. Anti-CD27L was found to improve disease and reduce autoantibodies in murine collagen-induced arthritis [54]. 

### 3.6. TNFSF10—TRAIL

TNF-related apoptosis-inducing ligand (TRAIL), also termed Apo2L, can be expressed by various cell types. It binds TRAIL receptors 1 to 4 and osteoprotegrin (OPG), inducing target cell apoptosis.

Only one report is available that describes TRAIL in PM, stating that many inflammatory cells are TRAIL positive [55]. TRAIL is expressed in the endomysial capillaries of healthy skeletal muscle and patients alike.

TRAIL potentially dampens autoimmune responses by silencing autoreactive T-cell populations and, therefore, could be beneficial to patients suffering from inflammatory diseases. A study showed that a blocking anti-TRAIL monoclonal antibody, on the one hand, exacerbates murine experimental autoimmune encephalomyelitis, while recombinant TRAIL, on the other hand, suppressed disease [56]. Thus, strategies delivering a soluble TRAIL equivalent may be effective in suppressing disease episodes. In addition, a human study identified TRAIL as a prognostic marker for IFN*β*-response in multiple sclerosis (MS). Upregulated concentrations of TRAIL in response to IFN*β* distinguished drug responders from nonresponders [57].

### 3.7. TNFSF11—RANKL

Receptor activator of NF-*κ*B ligand (RANKL), expressed on the membranes of T-cells, is also called TNF-related activation-induced cytokine (TRANCE), OPG ligand (OPGL), or osteoclast differentiation factor (ODF). Two putative receptors for RANKL have been proposed, being RANK and OPG. RANK is a transmembrane receptor present on DCs and T-cells. OPG is a soluble secreted decoy receptor for RANKL. RANKL acts in synergy with TNF*α*, activating a cascade of intracellular signaling events which lead to osteoclast activation.

In mice, RANKL mRNA is expressed in healthy skeletal muscle [58], which points to a role in normal muscle physiology. A study reported serum RANKL concentrations to be significantly higher and RANK levels to be significantly lower in juvenile DM than in healthy age-matched controls [59]. 

RANKL/RANK is of major pathophysiological importance in the bone and joint destruction associated with RA [59], where RANK expression appears to be limited to the sites of immune reaction. The development of compounds that mimic OPG action may prevent osteoclast-mediated bone loss in patients [60]. Denosumab (AMG-162), a monoclonal anti-RANKL antibody, is currently being tested for treating RA.

### 3.8. TNFSF13—BAFF and APRIL

B-cell activating factor (BAFF), also termed TNFSF13b or B-lymphocyte stimulator (BLyS), is expressed on the surface of monocytes, DCs, and activated T-cells. BAFF binds three receptors: transmembrane activator and calcium-modulating cyclophilin ligand interactor (TACI), BAFF receptor (BAFFR), and B-cell maturation antigen (BCMA). BAFF is crucial for B-cell maturation and survival and antibody production by plasma cells. In addition, BAFF regulates T-cell activation and differentiation. A proliferation-inducing ligand (APRIL) or TNFSF13 is homologous to BAFF and exists only as a soluble homotrimer. APRIL binds TACI and BCMA and is important for B-cell development and function [61].

Serological studies have shown that BAFF levels are significantly increased in DM patients [62] but not in PM/IBM [63]. BAFF levels in serum correlated with IL-7, IL-12, and CXCL10 [64] and with Jo-1 expression, supporting a role for BAFF in autoantibody production. In addition, BAFF transcripts were found markedly upregulated in muscle extracts from DM (12-fold), PM (14-fold), and IBM (21-fold) patients [65]. In DM muscle, BAFF localizes to the muscle fibers in perifascicular areas [66]. Interestingly, mononuclear cells infiltrating IM muscle express IFN*α* [67], a potent BAFF inducer. Serum APRIL levels were found unaltered in IM patients [64]. 

Blocking BAFF and APRIL potentially diminishes autoreactive B-cells, which would interrupt B-cell differentiation and prevent autoantibody production. Thus, BAFF and APRIL represent appropriate targets for intervention in autoimmune diseases with an important humoral pathogenic component. B-cells are especially associated with DM infiltrates, where IFN*α* expression could well be the trigger to activate autoantibody production. In addition, differentiated plasma cells can also be encountered in PM/IBM muscle samples [68].

The anti-BAFF monoclonal antibody belimumab has been tested in two phase III trials for the treatment of SLE. In both trials, belimumab met the primary endpoints, showing significant clinical improvement compared to standard of care alone. LY2127399, another BAFF neutralizing antibody, has entered phase II trials for RA. Atacicept, an Ig fusion protein of the extracellular domain of the TACI receptor that binds BAFF and APRIL, has currently reached phase II/III for treating SLE [69].

## 4. TNF Members Not Yet Explored in IM

### 4.1. TNFSF8—CD30L

CD30L is expressed on the membranes of activated T-cells, resting B-cells, and monocytes. Interaction with its receptor CD30, expressed on T-cells, and B-cells, leads to their proliferation and activation. In inflammatory diseases, CD30L/CD30 interactions seem to represent both deleterious and beneficial effects. A blocking monoclonal anti-CD30L antibody aggravates allograft rejection in mice by suppressing regulatory T-cell function [70], while soluble CD30-Ig fusion protein ameliorates murine experimental colitis through inhibition of Th17 responses [71]. Elevated levels of soluble CD30 have been observed in autoimmune diseases such as RA [72] and SLE [73].

### 4.2. TNFSF9—4-1BBL

4-1BBL is predominantly expressed on activated antigen presenting cells and interacts with the 4-1BB receptor expressed early and transiently on activated T-cells and on DCs. The 4-1BBL/4-1BB interaction is relevant to the pathogenesis of inflammatory disease. In sera of RA patients, soluble 4-1BB and 4-1BBL levels are increased and correlate with disease severity [74]. Treatment with an antagonistic anti-4-1BB antibody reduces severity of arthritis in animal models, ameliorating inflammation, and the associated B-cell responses [75].

### 4.3. TNFSF12—TWEAK

The multifunctional cytokine TNF-like weak inducer of apoptosis (TWEAK) triggers multiple and often seemingly conflicting cellular responses, which range from cell proliferation to cell death. Moreover, TWEAK signaling through the FN14 receptor [76] has an impact on normal muscle functioning. In vitro, TWEAK inhibits the differentiation of myoblasts to myotubes [77] and induces the expression of proinflammatory CCL2 [78]. TWEAK knockout mice exhibit augmented muscle tissue regeneration [79], while overexpression results in inhibited myofiber regeneration and increased expression of proinflammatory cytokines including TNF*α*, IL-1*β*, IL-6, and CCL2 [80–83]. The proinflammatory cytokines inducible by TWEAK are important regulators of IM [84], which warrants further exploration. Also, circulating TWEAK levels are significantly increased in other human autoimmune diseases such as MS and SLE [85]. A TWEAK-neutralizing monoclonal antibody ameliorates collagen-induced arthritis in mice, reducing serum and joint CCL2 levels significantly [86].

### 4.4. TNFSF14—LIGHT

Lymphotoxin-related inducible ligand that competes for glycoprotein D binding to herpes simplex virus entry mediator (HVEM) on T-cells (LIGHT), also termed LT*γ*, binds the receptors LT*β*R, HVEM, and the decoy receptor DCR3. LIGHT is expressed by activated T-cells and immature DCs, and is a potent T-cell costimulatory molecule [87] with profound effects on T-cell-mediated disease [88]. LIGHT enhances Th1-mediated immune responses and in vitro strongly induces CXCR3 ligands [89]. The predominance of Th1-mediated immunity and CXCL10 expression have been demonstrated in the IM [90]. In synovial tissues from RA patients, both LIGHT and its receptor HVEM are expressed by CD68+ macrophages, and their interaction induces the proinflammatory cytokines TNF*α*, IL-6, and IL-8 [91]. Blocking LIGHT activity significantly reduces graft versus host disease [92, 93].

### 4.5. TNFSF15—TL1

TNF-like 1 (TL1), also termed vascular endothelial growth inhibitor (VEGI), is expressed by macrophages, lymphocytes, and plasma cells. TL1 binding enhances the expression of IFN*γ* by T-cells [94] and induces apoptosis in endothelial cells. TL1 has been implicated in human inflammatory bowel disease, where it is found to be increased in macrophages, plasma cells and lymphocytes [95], and TL1 gene variants have been linked to disease susceptibility [96, 97].

### 4.6. TNFSF18—GITRL

Glucocorticoid-induced TNF receptor ligand (GITRL), also termed TNF-like 6 (TL6), is expressed by endothelial cells, DCs, macrophages, and B-cells. Its receptor GITR is present on naive, activated, and regulatory T-cells that, upon ligation, proliferate and produce cytokines. In RA, both GITR and GITRL are expressed in synovial macrophages that, in response to in vitro stimulation with an anti-GITR monoclonal antibody, produce TNF*α*, IL-6, IL-8, and CCL2 [98]. Agonistic anti-GITR monoclonal antibodies exacerbate joint inflammation and cytokine production [99].

## 5. Conclusions and Future Prospects

Oral corticosteroids are still standard treatment for DM and PM, but they come with serious side effect and incomplete treatment responses. Patients anxiously await more selective treatment options. Moreover, IBM patients do not respond to the immunosuppressive and immunomodulatory drugs currently available. A better understanding of the deleterious and beneficial effects of the different players that make up the IM muscle microenvironment is necessary to aid the development of novel routes for therapy. In addition, such knowledge could provide markers to distinguish potentially responsive from nonresponsive patients, better predicting the outcome of costly immune interventions.

**Figure 1:**
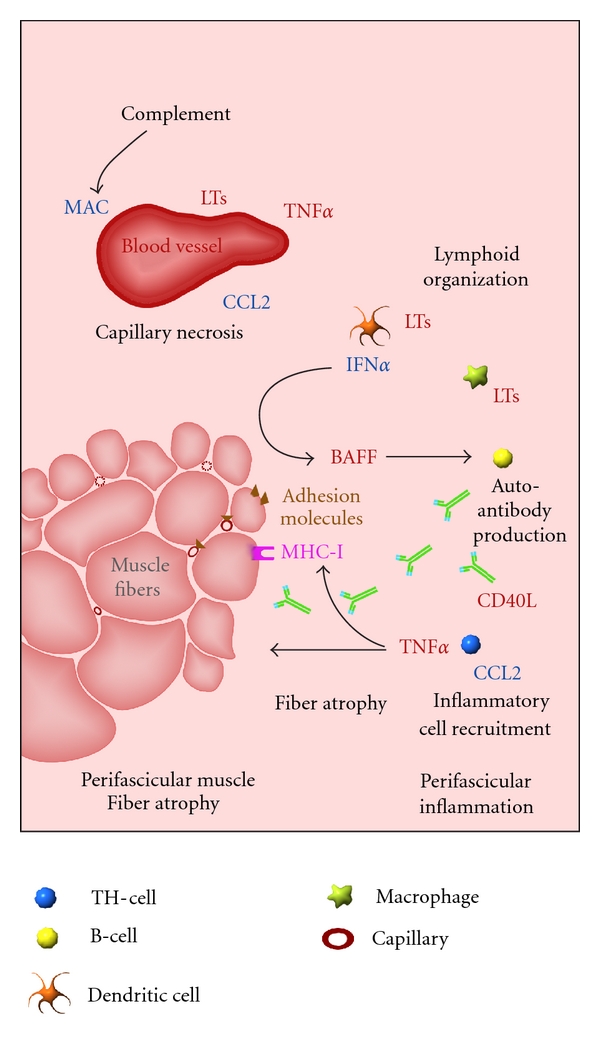
Potential roles for tumor necrosis factor cytokines in the muscle damage associated with dermatomyositis. Based on current knowledge, a model is developed describing the possible involvement of tumor necrosis factor (TNF) cytokines in the sustained inflammation in perifascicular regions of muscle. Activation of the complement system leads to membrane attack complex (MAC) deposition on blood vessels and subsequent necrosis. Endothelial monocyte chemoattractant protein-1 (CCL2) recruits inflammatory cells that accumulate and organize in perimysial areas. Lymphocytes organize into functional compartments and produce lymphotoxins (LTs), TNF*α* and CD40L, which further recruit responsive immune cells from the circulation, leading to the buildup of perimysial inflammation. Dendritic cells (DCs) produce IFN*α*, which stimulates muscle fibers to secrete B-cell activating factor (BAFF). The latter activates B-cells that, in response, begin to produce autoantibodies. TNF*α*, mostly produced by Th-cells, provokes muscle fiber atrophy and stimulates major histocompatibility complex I (MHC-I) and expression of adhesion molecules.

In this paper, we summarized current knowledge on the involvement of the TNF superfamily of cytokines in IM, finding ourselves humbled by the lack of data regarding some of them. The TNF cytokines represent plausible therapeutic targets for the IM, as they are regulators of the complex inflammatory cascade that leads to sustained inflammation. For PM and IBM, limited information is available for TNF cytokines other than TNF*α*. However, for DM, a picture is slowly emerging in which TNF cytokines no doubt play a crucial role. Based on current knowledge, we developed a model describing the TNF-mediated sequence of events that lead to the characteristic muscle damage, being blood vessel loss, perifascicular muscle fiber atrophy, and inflammation (Figure 1). 

Selectively targeting individual TNF members provides new promises and opportunities to develop more efficacious therapies for IM while avoiding the toxicity seen in existing systemic anti-inflammatory therapeutics.

## Figures and Tables

**Table 1:** Tumor necrosis factor inhibitors for treating inflammatory myopathies: published trial results for infliximab and etanercept.

Compound and treatment regimen	Diagnosis/patients continued to end point	Follow-up time	Clinical outcome at end point	Reference
infliximab 6 mg/kg 4-weekly or more frequent	R-JDM/5	32 to 130 weeks	I (5/5)	[19]
infliximab 10 mg/kg (week 0, 2, 6, 14)	R-DM/1 R-PM/4 R-IBM/4	16 weeks	NC (1/1) I (2/4) W (2/4) I (1/4) NC (3/4)	[20]
infliximab 10 mg/kg (week 0, 2, 4)	R-DM/1 R-PM/1	12 weeks	I (1/1) I (1/1)	[21]
infliximab 10 mg/kg (week 20) infliximab 10 mg/kg (week 14, 18, 22)	R-DM/1 R-PM/1	66 weeks	I (1/1) I (1/1)	[22]
infliximab 10 mg/kg (week 0, 2, 6, 14, 22)	PM/2	26 weeks	I (2/2)	[23]
infliximab 8 mg/kg (week 0, 2, 6)	R-DM/1	6 weeks	I (1/1)	
infliximab 10 mg/kg (week 0, 2, 4, 6, 9)	R-PM/1	69 weeks	I (1/1)	[24]
infliximab 3 mg/kg (week 0, 2, 6, every 8) and etanercept 25 mg twice weekly	R-DM/1 R-PM/2	36 to 96 weeks	PR (1/1) I (2/2)	[25]
etanercept 25 mg twice weekly	R-DM/1	56 weeks	I (1/1)	[26]

Abbreviations: dermatomyositis (DM), improved (I), inclusion body myositis (IBM), juvenile DM (JDM), no change (NC), partial response (PR), polymyositis (PM), refractory DM/PM/IBM (R-DM/PM/IBM), worsened (W).
